# Technology-Based Interventions, Assessments, and Solutions for Safe Driving Training for Adolescents: Rapid Review

**DOI:** 10.2196/11942

**Published:** 2019-01-24

**Authors:** Emre Sezgin, Simon Lin

**Affiliations:** 1 Research Information Solutions and Innovation, The Research Institute Nationwide Children's Hospital Columbus, OH United States

**Keywords:** adolescent health, assessment, driving safety, teen driving, technology-based intervention

## Abstract

**Background:**

Safe driving training for adolescents aims to prevent injury and promote their well-being. In that regard, information and communication technologies have been used to understand adolescent driving behavior and develop interventions.

**Objective:**

The purpose of this review is to explore and discuss existing approaches to technology-based driving interventions, driving assessments, and solutions in the literature.

**Methods:**

We searched the Web of Science and PubMed databases following a review protocol to collect relevant peer-reviewed journal articles. Inclusion criteria were (1) being published in the English language, (2) being published in a peer-reviewed journal, (3) testing the driving behavior of teens with technology-based intervention methods, and (4) being published between January 2000 and March 2018. We appraised the articles by reading their abstracts to select studies matching the inclusion criteria and reading the full text of articles for final refinement.

**Results:**

Initial keyword searches on technology-based solutions resulted in 828 publications that we refined further by title screening (n=131) and abstract evaluation against inclusion criteria (n=29). Finally, we selected 16 articles that met the inclusion criteria and examined them regarding the use of technology-based interventions, assessments, and solutions. Use of built-in tracking devices and installation of black box devices were widely used methods for capturing driving events. Smartphones were increasingly adapted for data collection, and use of gamification for intervention design was an emerging concept. Visual and audio feedback also were used for intervention.

**Conclusions:**

Our findings suggest that social influence is effective in technology-based interventions; parental involvement for promoting safe driving behavior is highly effective. However, the use of smartphones and gamification needs more study regarding their implementation and sustainability. Further developments in technology for predicting teen behavior and programs for behavioral change are needed.

## Introduction

### Background

The US National Center for Health Statistics reported that 73% of unintentional injury deaths among teenagers in the United States were caused by motor vehicle traffic incidents over the years 1999-2006 [1]. Motor vehicle crashes continue to be one of the leading causes of deaths among teenagers, and most incidents were attributed to risky behavior established during childhood [2]. Teen drivers have crash rates almost 3 times higher per mile driven than drivers 20 years and older [3]. Immaturity leads to speeding and other risky habits, and inexperience means teen drivers often do not recognize or know how to respond to hazards [4]. This issue highlights the question that has been raised by the US National Research Council, Institute of Medicine, and Transportation Research Board [5]: “What are the best ways to influence teens’ behavior?”

### Current Practices

To answer the question, several solutions have been proposed by national organizations and associations in the United States. One major approach that is widely implemented is to improve driver education and training programs [6,7]; however, the effectiveness of such programs needs further evidence [6]. Another recent approach is the use of technology to monitor driving. The US Insurance Institute for Highway Safety reported that teens who used in-vehicle monitoring devices showed less risky behavior than unsupervised teens [8]. However, the technology-based intervention is only effective if parents are able to review the feedback and talk about it with their teen. In another practice by the US Governors Highway Safety Association, dashboard cameras for monitoring driving activity are used for postdriving training [4], but it was found not feasible due to the costs of equipment purchase and installation. The Minnesota Department of Transportation implemented a smartphone-based driving support system and tested it with the participation of teen-parent pairs [9]. Teens found the system helpful for complying with the rules and reducing risky driving behavior. However, the ability of teens to adapt to the system warnings and parents’ concerns about their teens’ privacy were major issues.

### In-Vehicle Technologies

In-vehicle technologies include dedicated information system tools to understand driving conditions, environment, and behavior. These technologies can be stand-alone systems (black box) or integrated with other technologies such as mobile devices. The purpose of in-vehicle technologies is to create a real-time digital footprint of a driving event. The components of an in-vehicle system can be smartphones, communication tools (eg, short message service, email, or external information channels), and vehicle diagnostics to collect relevant information. For instance, the in-vehicle technology Foot-LITE uses real-time information on road conditions and vehicle operation, and collects data using a camera, a 3-axis accelerometer, and a global positioning system [10]. It connects to the vehicle with an onboard diagnostics (OBD-II standard) port and processes the data using an onboard processing unit (TRW Limited engine control unit). The system provides feedback on driving behavior, such as a lane departure warning, via a smartphone app. The advanced driver-assistance system (ADAS) is another widely used in-vehicle technology. ADAS has been used to adjust vehicle operation to improve safety and driving. It is an integrated system designed to understand real-time events and alert drivers to avoid collisions. The extent of the capabilities of ADAS may vary, but some examples are lane departure warning, blind-spot warning, adaptive lighting, and adaptive cruise control that autoaccelerates and autobrakes in traffic. Table 1 presents the information that can be potentially collected using in-vehicle technologies.

**Table 1:** Information collection and required hardware for in-vehicle technologies.

Data type	Source	Hardware
Weather conditions	National weather API^a^ service	Wireless internet connection, modem
Road type (residential, city, rural)	Map API service	Wireless internet connection, modem
Traffic light status	Camera	Smartphone, external hardware
Traffic sign detection	Camera	Smartphone, external hardware
Lane-marking detection	Camera	Smartphone, external hardware
Traffic condition	Web source via traffic API service	Wireless internet connection, modem
Traveling distances	GPS^b^	Smartphone, black box
Changes in velocity	GPS, accelerometer	Smartphone, black box
Changes in acceleration	Accelerometer, gyrometer	Smartphone, black box
Changes in geolocation	GPS	Smartphone, black box
Heart rate, electrocardiogram	Monitoring sensor	Smartwatch
Seatbelt	Built-in sensor	External hardware
Light exposure	Camera, light sensor	Smartphone, external hardware
Accident detection (rollover and impact)	Accelerometer, gyrometer, magnetometer	Smartphone, black box
Acceleration, braking, and cornering behavior	Accelerometer, GPS, gyrometer	Smartphone, black box
Following distance	Camera, infrared sensor	Smartphone, external hardware
Driver identification	Camera	Smartphone, black box
Traveling pattern	GPS, magnetometer	Smartphone, black box

^a^API: application programming interface.

^b^GPS: global positioning system.

### Objectives

Even though there is no “gold standard” for solving the safety issues of young drivers, the scientific quest for seeking solutions with technology-based interventions has been advancing. In that regard, some studies have revealed novel technologies and methods for increasing driving safety and awareness among teens. We believe that understanding these approaches would be helpful for identifying effective implementations of technology-based interventions. We sought to review the literature on the effect of technology-based interventions, assessments, and solutions on adolescent driving behavior. Therefore, we aimed to (1) explore the technology-based approaches reported in the literature, (2) discuss their methods and findings, and (3) suggest alternative approaches in the light of the findings.

## Methods

We limited the scope of the literature search to peer-reviewed journal articles indexed in the Web of Science and PubMed databases, which provide access to scientifically rigorous studies in reputable and indexed journals. Inclusion criteria were (1) being published in the English language, (2) being published in a peer-reviewed journal, (3) testing the driving behavior of teens with technology-based intervention methods, and (4) being published between January 2000 and March 2018. Our search strategy was to (1) identify search keywords, (2) refine the selection of journal articles, (3) read abstracts to select studies matching the inclusion criteria, and (4) read the full text of articles for final refinement. We searched the databases using the following combinations of keywords: “teen” OR “adolescent” OR “young” AND “driving” OR “driver” AND “technology” OR “smartphone” OR “phone” AND “vehicle” AND “prevention” OR “intervention”.

We extracted data into a predesigned Excel spreadsheet form (Microsoft Office 2016; Microsoft Corporation). The form included study title, year, journal, scope of the study, method, sample characteristics and size, and study findings. We performed a qualitative synthesis to descriptively synthesize the data.

## Results

### Search Results

**Figure 1:**
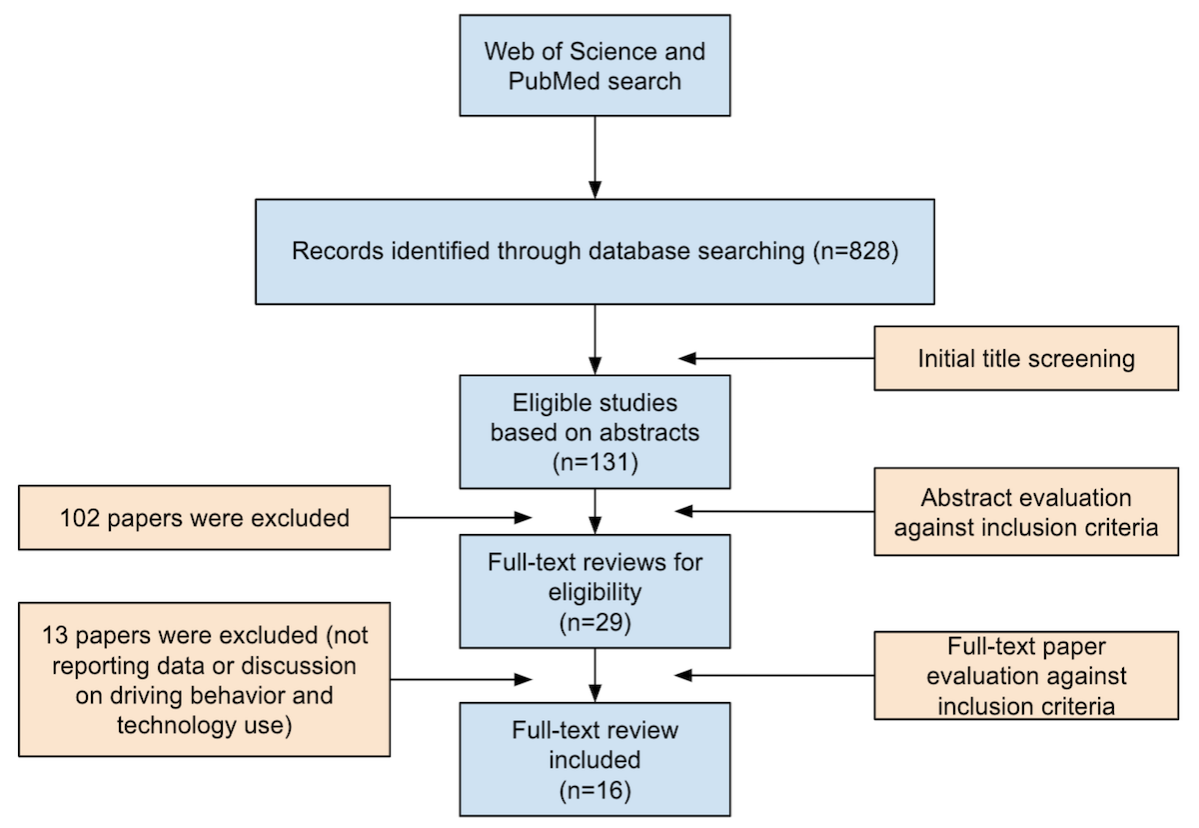
Review flow diagram.

We completed the search by March 2018. We initially identified 828 records (147 from Web of Science and 681 from PubMed), which we further refined by title screening (n=131) and abstract evaluation against the inclusion criteria (n=29). After full-text review, we identified 16 studies [11-26] that focused on the driving behavior of teens and promoting behavior change with technology-based interventions (Figure 1).

### Technologies to Improve Teen Driving Safety

Our research results fell into 3 categories of technology used for teen driving safety: (1) in-vehicle technologies (using built-in tracking devices in the car and an installed black box), (2) smartphones (using apps and the sensors of a smartphone), and (3) gamification (an extension of smartphones used to increase compliance and sustainability of safe driving).

#### In-Vehicle Technologies

##### Technology-Based Interventions and Assessments

The effectiveness of in-vehicle technologies for teen driving safety has been evaluated through longitudinal tests and randomized trials. In accordance with the reports mentioned in the introduction, parental involvement in combination with in-vehicle technology was identified as a highly effective intervention with the in-vehicle technologies. Farah et al [11] conducted a longitudinal study with the participation of 242 families of young male drivers. In-vehicle data recorders were placed in the cars for 12 months during a teen’s first year of driving. The first 3 months consisted of the teen driving with a parent or family member, and the remaining 9 months consisted of the teen driving solo. There were 4 study groups based on family feedback and guidance on parental involvement. Drivers in the groups who received family feedback with or without guidance on parental involvement had lower event occurrence rates than did the control (no-feedback) group during the solo-driving phase. This finding indicated that early intervention using the combination of in-vehicle technology and family/parental involvement had a lasting effect on teen driving behavior. Similarly, Musicant and Lampel [12] recruited 32 young drivers to test the effectiveness of an intervention using an in-vehicle recording mechanism for capturing driving behavior from 113 to 237 days. The study had 2 phases, with and without feedback to families and teens. During the feedback phase, the occurrence of risky events was reduced. Farmer et al [13] tested 4 parameters of teen driving behavior using a driving detection system (acceleration, braking, speeding, and seat belt use). The groups had different interventions, such as receiving alert sounds for speeding and not using a seat belt, and parental access to Web-based feedback. This study found that the use of seat belts increased as the violations were reported to parents, compared with in-vehicle alert sounds. Speeding violations were decreased with in-vehicle alerts. Finally, technology-based monitoring of teen driving was able to reduce the incidence of risky behavior. However, the child-parent relationship and dynamics influenced the effectiveness of the intervention.

##### Impact of Social Influence and Parental Involvement

Simons-Morton et al reported in 2 studies [14,15] the implications family or parental involvement on teen driving behavior. The first study [14] tested the impact of an in-vehicle safety monitoring system on 2 groups of teens. One group received immediate feedback from the system about risky driving, while the other group received weekly reports and family access to driving scores in addition to the immediate feedback. The intervention that included parental involvement was more effective than the use of the technology alone. The follow up study [15] identified risk factors in teen driving. The participants were observed during the first 18 months after licensure, and data were collected on driving behaviors (eg, acceleration, braking, and location) via video, images, and periodic surveys. Saliva swabs were collected and tested for stress-induced compounds, and distraction and driving skills were scored. The findings suggested that crash and near-crash risks were almost 4 times higher in teens during the first 18 months after licensure than among adults. The authors argued that social norms strongly influence driving behavior: the risk of crash was higher for teens driving alone than while driving with passengers.

McGehee et al [16] used an event-triggered device to capture driving data, and audio and video feeds from the driver to measure risky behavior. Participants were observed using a 3-phase design to measure changes in driving behavior between the no-intervention and intervention (immediate feedback and feedback by parent and teen mentoring sessions) phases. Use of the event-triggered video system with weekly feedback and video review involving parents reduced the unsafe driving behavior of teens. A previous study by Carney et al [17] supported this finding. A similar 3-phase design with in-vehicle monitoring technology was implemented with the participation of 18 young drivers. Intervention with visual feedback and weekly event reports to teens and parents reduced risky driving behavior by 61%. To understand parental guidance for newly licensed young drivers, Prato et al [18] investigated the behavior of teen-parent pairs. They recruited 62 families; vehicle monitoring systems were installed in their cars, and driving behaviors were monitored (the first 3 months was the accompanied-driving setting, and the next 9 months was the solo-driving setting). Findings suggested that risk-taking behavior could be influenced by the driver’s sex, observations of parental driving, sensation-seeking tendencies, duration of supervised driving, and the level of parental involvement in monitoring the teens.

##### Uptake Challenges

Interviews also revealed latent facts about integrated technology use. Gesser-Edelsburg and Guttman [19] interviewed 2 groups of teens: 1 group used an in-vehicle driver monitoring system (n=26) and 1 did not use a monitoring system (n=111). Findings suggested that the system may have had adverse effects on perceiving technology as a solution because it replaced parental accompaniment (as a tool for monitoring, punishment, and violation of privacy). However, the teens had a positive attitude toward the system for being an objective and credible source of driver behavior and for helping to improve driving skills. The authors argued that there is a need to create a support system of professionals for teens and parents, and that the technology should have the role of facilitator of the intervention. Weiss and colleagues’ [20] focus group interviews demonstrated that teens were comfortable with the technology and familiar with its limitations. Thus, they were not willing to have interference by the system (ADAS) while driving to more naturalistically develop their skills.

To understand the perspective of parents, Guttman et al [21] interviewed parents of young drivers regarding the use of in-vehicle monitoring technologies. The participants addressed slightly different issues, in that monetary cost and security concerns were the main factors discouraging installation of the technology. Young drivers receiving feedback and monitoring of driving were the main motivating factors to install the technology. Furthermore, the parents expected to take this step in the early stages of driving. Promotion of the technology would require more incentives and lower cost. Moreover, developing a clear policy on security and privacy about driving data and legal implications, addressing young drivers’ privacy concerns, and providing resources for parents to guide their kids for safe driving were identified as critical aspects for implementing integrated technology approaches. In terms of policy implications to promote the uptake of these technologies, McGehee et al [16], Simons-Morton et al [14], and Carney et al [17] reported the significance of parental involvement in supervision in technology and use of graduated driver licensing.

#### Smartphone Use

Use of smartphones for teen driving safety is a relatively new concept. The increasing capability of smartphones, low cost of access, and higher accuracy in capturing events have promoted the use of smartphones for quantifying driver behavior. To understand the effect of safe driving apps, Creaser et al [22] tested the effect of a phone blocking app in 3 groups. The first group used the blocking app. The second group used the same app and parents received reports of risky events. For the third group, the control group, driving behaviors were just observed. Even though the study data revealed low use of the phone during driving, self-reported data showed that teens were able to find a way around blocking, and use of a mandatory setting would not have been helpful in the long run.

The use of safe driving apps, as well as the influence of the social environment, were investigated by Musicant et al [23]. They conducted a longitudinal study with young scouts and cadets to investigate the effect of a safe driving app, which was promoted with the use of a group incentive scheme. The app recorded events on each trip and provided feedback and a score based on driving behavior (speed, acceleration, braking, and cornering). The study demonstrated that young people may act for the benefit of the group. Low-cost and group incentive schemes could motivate young drivers to use the safety app. However, the effect may only have been temporary; lack of incentives, short trips, battery consumption, and forgetting to enable the app were the reasons given for not using the app. Similarly, Kervick et al [24] investigated the willingness of young drivers to use a smartphone-based driver support system. The perception of what the teen would gain from using the system and the influence of social environment on using the system were the factors determining the intention to use versus actual use.

#### Adaptation with Gamification

With the integration of smartphones in driving safety, gamification emerged as an intervention for behavioral change. In that regard, Steinberger et al [25] investigated several game concepts with young male drivers to encourage safe driving. Drivers tested the smartphone games (mounted on the dashboard) while driving via a drive simulator. Results showed that engagement was associated with economic concerns (fuel consumption) and anticipatory driving (what was ahead). In addition, participants expected a degree of challenge from the game to make driving fun, interactive with others, and personalizable (based on different characteristics and patterns). The authors also studied the effect of gamification on reducing driving boredom. Steinberger et al [26] tested a mobile game concept to encourage anticipatory driving by detecting speed limits and changes. They recruited 2 groups of teens as control and intervention groups to use a drive simulator. Driver data (eg, lane position, speed, video, and physiological measures) and subjective experience data (eg, surveys about boredom intensity, arousal, and perceived driving performance) were collected. Results showed that gamified intervention may reduce unsafe driving by reducing driving boredom. However, visual cues can increase cognitive workload and thus cause slower reaction times to driving events. The authors also indicated that physiological measures can help to identify driving boredom events.

Table 2 [11-26] summarizes the literature findings and provides a broader look at the study methods, significant findings, and barriers to technology use.

**Table 2:** Literature summary grouped by the type of technology used.

Study		Country	Method	Sample and size	Significant findings for technology use	Identified barriers to technology use
**In-vehicle technologies**						
	McGehee, 2007 [16]	United States	Driving data analysis (technology used: DriveCam)	26 teens (16-17 years old)	Technology with periodic feedback and parental involvement were effective in reducing unsafe driving.	N/A^a^
	Musicant, 2010 [12]	Israel	Driving data analysis	32 young drivers (17-24 years old)	Availability of feedback reduced event frequency by 50%,	N/A
	Carney, 2010 [17]	United States	Driving data analysis (technology used: DriveCam)	18 teens (16 years old)	Intervention with visual feedback and weekly reports and videos to teens and parents increased safe driving.	N/A
	Prato, 2010 [18]	Israel	Driving data analysis and survey	62 teen-parent pairs	Different sexes exhibited different risky behaviors; Tendency to seek sensation affects risky driving; Driving behavior of parents, duration of supervised driving, and level of parental monitoring influenced risky behavior.	N/A
	Farmer, 2010 [13]	United States	Driving data analysis	85 teens (16-17 years old)	Reinforcement from parents was necessary for sustainable safe driving; Push notifications (emailing report cards and personalized feedback) were more effective than pull notifications (website access).	Alerts can be annoying; Too much information provided could be discouraging for parents
	Guttman, 2011 [21]	Israel	Interview	906 parents of young drivers (17-24 years old)	Early stages of driving were considered a better time for installing the technology; Financial benefits and environmental considerations were perceived as incentives; Security of data and privacy of teens were common concerns; Technology may promote parent-teen driver communication; Parents should have access to monitoring data.	Cost; Security and privacy concerns; Confronting the young driver
	Simons-Morton, 2013 [14]	United States	Driving data analysis and survey (technology used: DriveCam)	90 parent-teen couples (~16 years old)	Parental involvement increases effectiveness.	N/A
	Simons-Morton, 2015 [15]	United States	Driving data analysis and survey	42 teens (~16 years old)	Social norms were important in risky behavior; Driving alone was riskier than with passengers.	N/A
	Gesser-Edelsburg, 2013 [19]	Israel	Interview	137 teens (15-18 years old)	In-vehicle technology was an objective and credible source for driving; Replaced the role model of parents with objective feedback from the device.	Trust issues within parent-teen relationship; Invasion of privacy; Stress from parental punishment based on feedback; Doubts about the technology improving driving skills
	Farah, 2013 [11]	Israel	Event frequency analysis (technology used: GreenRoad Tech)	212 teen-parent pairs	Periodic driving feedback, parental involvement, and guidance were effective in reducing risky driving.	N/A
	Weiss, 2018 [20]	United States	Interview (technology used: advanced driver-assistance system)	24 teens (16-19 years old) and 12 parents	Teens were knowledgeable about and comfortable with the technology; Teen and parents preferred using a non–advanced driver-assistance system car to improve driving skills.	Teens are skeptical about abilities of the technology, knowing its limitations; The idea of giving control to a “machine” is not positively perceived
**Smartphone**						
	Musicant, 2015 [23]	Israel	Interview and survey	24 scouts and 22 cadets (17-19 years old)	Group incentives and low cost improved uptake of in-vehicle technology.	Forgetfulness; Battery consumption; Lack of incentives
	Creaser, 2015 [22]	United States	Survey	274 teens and 272 parents	The blocking app could be effective for new drivers; Parental involvement with the app increased the effectiveness.	Bypassing the app or using a friend’s phone
	Kervick, 2015 [24]	Ireland	Survey	333 teens (18-24 years old)	Perceived gains from use of the app and social influence affected acceptance of the driving support app.	N/A
**Gamification with smartphone**						
	Steinberger, 2017 [25]	Australia	Design analysis and interview	24 young men (~20 years old)	Economic and anticipatory driving were engaging; Drivers expected a challenge from the game; Interaction with others was important; Personalization was desired	N/A
	Steinberger, 2017 [26]	Australia	Driving data analysis and interview	32 young men (18-25 years old)	Ambient feedback with colors was useful.	Instant visual feedback can be distracting; Screen positioning can be distracting

^a^N/A: not available.

## Discussion

In the light of the findings about the effects of in-vehicle technologies on teen driving behavior, we propose several implications and suggestions that could provide a basis for future development of interventions.

### Parental Involvement in Technology Effectiveness

The findings indicate that in-vehicle technologies are useful for assisting teens with safe driving behaviors; parental involvement along with technology-based feedback has an even stronger influence on development of safe driving skills. Technology is viewed as a double-edged sword because it creates both an opportunity for and a barrier to the parent-teen relationship. On the one hand, it can be used to develop trust between parents and teens by providing an objective source for driving and feedback; however, teens can also perceive in-vehicle technologies as a sign of parental distrust. Therefore, when promoting the technology, the purpose of its use should emphasize the benefit to teens.

As reported in most of the studies, implementation of the technology should consider parents’ attitudes toward the use of technology as well. In terms of parenting style, teens with highly involved parents (providing a level of support, but highly involved with rules and monitoring; ie, they are authoritative) are less likely to demonstrate risky (deliberate risk-taking) behavior and be more compliant with rules [27]. Similarly, involving adult passengers as well as receiving postdriving feedback with parental involvement could be effective in teens’ development of safe driving skills [28]. While studies reported that parents have a major influential role in driving, the unwillingness of a teen’s parent to implement in-vehicle technology or to be involved with the teen may be a barrier to the teen’s development of safe driving skills [29]. In that regard, parents’ personal traits and parent-teen dynamics, as well as environmental and living conditions, should be regarded as determining factors for the effectiveness of technology-based interventions. In addition, timely interventions, providing continuous feedback for parents and teens, and providing education resources and incentive mechanisms for parents and teens should be considered as key success factors for implementing technology-based interventions.

### Extending Research and Gaining Evidence via Smartphone Use

Smartphone use is a promising means of delivering technology- based intervention for teens, as 2016 statistics from the Pew Research Center reported that 92% of young adults had smartphones [30]. Target audiences can be reached via smartphones at low cost, without interference with daily life, and without the need for high user involvement in data collection. Some studies reported that the use of smartphones has the potential to be attractive to teens because of the availability of timely feedback, interaction via apps, integration with other apps, and timely connection with users, as well as by offering incentives and the potential for social connection.

However, in terms of smartphone-based interventions and assessments to prevent risky teen driving, more evidence-based findings are needed, especially under real-world conditions. Because only a few studies about smartphone-based interventions for teen drivers have been conducted, the relevant literature about the general population can be used to design the methodology for developing interventions for teens. For example, the most desirable smartphone features were text blocking, collision warning, voice control, and driving data recorders for the general population [31]. Thus, the apps being used by adult drivers might be adapted and tested with teen users. In another case, a collision warning app effectively reduced event occurrence for adults [32], and feedback helped to improve driving efficiency and driving behavior for safe following distance [10]. The extension of these studies to teen driving safety and behavior change would help fill the knowledge gap. However, there could be challenges in adapting these existing methods for teens in terms of deciding on an intervention method, such as use of unobtrusive technology (which is more acceptable) versus intrusive technology (which is less acceptable) [28]. Battery consumption and incentivization are other challenges to overcome for ensuring teens’ continuous use of smartphone-based solutions.

### Gamification in Play

Gaming stimulates self-efficacy and reward mechanisms to promote particular behaviors. In the health care literature, gamification was reported to have positive effects on behavioral and cognitive outcomes [33]. Therefore, gamification could promote healthy driving behavior in adolescents via intrinsic motivation. The method also is highly accessible on mobile devices, is cost effective, and fits into the current lifestyle.

Based on the findings, use of a gaming approach could be effective in promoting behavioral change for safe driving. However, there is a lack of evidence of the effectiveness of design and in real-world driving implementations. Users expect a challenge, interaction, social connection, and personalization from a game, and it is challenging to fulfill these needs without causing a distraction. To further implement gamification without distracting the driver, postdriving feedback is suggested instead of feedback during driving. In that regard, drive scoring, leaderboards, and achievement badges could be used as feedback mechanisms. To assess the effectiveness of gamification in real-world implementation, wearables and biometric measures may provide feedback and observations on driving behavior change [34]. However, gamification has its own risks. Designers should note that increasing competition, design, and task evaluation issues may have adverse effects on behavior [35].

Regarding the extent of the research on in-vehicle technologies, smartphones, and gamification, the literature has presented more evidence of in-vehicle technology use with parental involvement. Studies of smartphones and gamification as safe driving interventions have been limited because they are relatively new concepts, and their effectiveness has been tested mostly under controlled environments.

### Further Suggestions on Technology-Based Intervention Developments

The literature discussed in this study suggests that the capability of current technologies and their adaptation for effective use are increasing. However, the element that has not been discussed but is significant for long-term impact is the use of big data. In-vehicle technologies and smartphones have been extended to collect aggregated driving data for better quantifying driving behavior, such as understanding driver behavior with pattern recognition for identifying aggressive driving [36], and identifying driver behavior features for better feedback via machine learning [37,38]. Specifically, the ability of smartphones to collect data is as good as that of advanced in-vehicle technologies [39], and smartphones have the potential to provide further evidence of effectiveness of interventions and assessment in the long term.

The literature also lacks evidence of the long-term impact of technology-based interventions. Thus, in addition to the technical capabilities, a deeper look into multilevel influences (eg, the sociotechnical perspective, social determinants of health) on teen driving behavior would also contribute to the design of interventions. Furthermore, security of driving data, privacy, stress (teens being punished for bad driving and parents wanting to avoid confrontation), trust issues, cost of implementation, and lack of incentives were observed as the major barriers to use of the technology. Therefore, the design of digital driving behavior change intervention programs may benefit from considering the engaging factors, risk factors, and protective factors for teens; developing communication methods; evaluating teen driver behavior; monitoring progress; and ensuring compliance with ethics, regulations, and information governance [40,41].

### Limitations

This review was limited to providing insight on in-vehicle technologies and intervention for teens based on the literature available in Web of Science and PubMed within our selection criteria. In addition, the study did not include research on driving distraction but focused on technology-based detection and intervention for injury prevention purposes. We listed the findings based on the technology being used, but we did not break down the findings to present method categorizations or the level of teens’ learning progress (eg, graduated driver licensing level, early-period or late-period novice learners). Similarly, the review did not address regional or national policies and regulations for transportation and driving. Thus, readers should consider regional differences while interpreting the findings.

### Conclusions

We reviewed the effects of technology-based interventions on adolescent driving behavior. We discussed in-vehicle technology and smartphone-based approaches and reported significant findings and observations. Finally, we provide suggestions for implementations and implications for further research. To our knowledge, there have been no literature reviews on teens and smartphone use and gamification of on-road driving. However, teen crash risks [42], distraction from mobile technology [43], effect of distraction on driving [44,45], and prevention of cell phone–based distractions [46] have been reviewed. This review extends the literature by filling in the gap in knowledge of technology-based intervention methods.

The study can be expanded with inclusion of other languages and databases. In that regard, we suggest including meta-analysis of trial studies with in-vehicle technologies in future work. Additional experimental studies on smartphones and gamification approaches would be useful to identify intervention methods, design requirements, and effectiveness of these new methods.

## Abbreviations

ADAS: advanced driver-assistance system
API: application programming interface
GPS: global positioning system
